# Lay Theories About Whether Emotion Helps or Hinders: Assessment and Effects on Emotional Acceptance and Recovery From Distress

**DOI:** 10.3389/fpsyg.2020.00183

**Published:** 2020-02-18

**Authors:** Melissa M. Karnaze, Linda J. Levine

**Affiliations:** Department of Psychological Science, University of California, Irvine, Irvine, CA, United States

**Keywords:** theories of emotion, emotion regulation, emotional experience, acceptance, suppression

## Abstract

This investigation examined how people’s beliefs about the functionality of emotion shape their emotional response and regulatory strategies when encountering distressing events. In Study 1, we present data supporting the reliability and validity of an 8-item instrument, the Help and Hinder Theories about Emotion Measure (HHTEM), designed to assess an individual’s beliefs about the functionality of emotion. Participants who more strongly endorsed a Help Theory reported greater wellbeing, emotional acceptance, and use of reappraisal to regulate emotion. Participants who more strongly endorsed a Hinder Theory reported less wellbeing and more expressive suppression and substance use. In Study 2, we demonstrate that encouraging participants to view emotion as helpful affected their physiological and regulatory response to a distressing event. Participants in the Help Theory condition showed greater physiological reactivity (SCL) during a distressing film than control participants but were more accepting of their emotional response. Shortly after the film, SCL decreased for participants in the Help Theory condition. Compared to control participants, they engaged in less suppression and reported less lingering effect of the film on their mood. Together, these studies suggest that people’s theories about the functionality of emotion influence their reactivity, the strategies they adopt to regulate emotion, and their ability to rebound after distressing events.

## Introduction

Disney’s 1943 cartoon film, “Reason and Emotion,” depicted emotion as a caveman living in the brain alongside reason, a modern businessperson sporting a suit and glasses. The film aimed to promote U.S. support for World War II but it also had a broader message. Reason should be in the driver’s seat with emotion strapped firmly in the back. Even today, popular culture and the media often portray emotion as the antagonist of reason, and convey the notion that people make wiser decisions unhindered by sentiment (Lutz, 1986; Parrott, 1995). Yet, popular culture also extolls the virtues of emotion. Emotion makes us fully human and gives life meaning. It motivates people to take important action and persevere in the pursuit of their goals. It provides a primary means of relating to others and motivates care for others (Lutz, 1986).

This dual perspective about whether emotion helps or hinders is also salient in academic theory and research. Economists and affective scientists argue that emotions are essential for guiding cognition and behavior (e.g., Simon, 1967; Frijda, 1994; Clore, 2011). Evolutionary psychologists argue that emotions evolved to help ancestral humans solve recurring problems such as overcoming goal obstruction (anger), avoiding pathogens (disgust), adjusting to loss (sadness), and finding a mate (desire; e.g., Tooby et al., 2008). Though emotions likely promote survival and reproductive fitness, this is not to say that any given emotional experience is helpful at any time. Researchers also recognize that, when too intense, too frequent, or inappropriate, emotion can interfere with effective decision-making, impede goals, cause added distress, and contribute to mental health problems (Kring, 2008).

Given the salience of competing perspectives on the functionality of emotion in popular culture, the media, and scholarship, surprisingly little is known about the extent to which lay people view emotion as helpful or harmful. We also know little about the consequences of these views for people’s emotional experience and wellbeing. Thus, this investigation presents a measure designed to assess how much people view emotion as helping them or getting in their way. We also assess whether endorsing a Help or Hinder Theory about emotion has implications for people’s emotional and regulatory responses and recovery from distressing events.

### Lay Beliefs About the Functionality of Specific Emotional States and Features

Researchers have examined the extent to which people view specific emotional states and features as helpful or harmful (e.g., Chow and Berenbaum, 2012; Manser et al., 2012). In one study, participants reported their emotional experience in daily diaries. Those who valued negative emotions (e.g., anger, nervousness) showed weaker links between the negative emotions they experienced day-to-day and poor psychosocial functioning and physical health (Luong et al., 2015). Other studies have shown that inducing positive beliefs about the functionality of specific emotional states (e.g., anxiety) or features (e.g., physiological arousal) promotes recovery from stressful situations (Low et al., 2008; Jamieson et al., 2010, 2012; John-Henderson et al., 2015), and wellbeing (Chow and Berenbaum, 2016). The fact that negative emotions can be viewed as useful shows that people’s beliefs about the functionality of emotion do not simply reflect how they want to feel (Chow et al., 2015), or how pleasurable they perceive certain feelings to be (Netzer et al., 2018). In general, then, valuing specific emotions, or specific features of emotion, tends to be associated with better outcomes than viewing them as dysfunctional (Brooks, 2014; De Castella et al., 2014; Veilleux et al., 2015; Crum et al., 2017; Ford et al., 2018).

Western media and discourse, however, often portray emotion overall as either helpful or a hindrance. Lay people may also hold views about the functionality of emotion generally. This is not to say that people believe emotions are always adaptive or always maladaptive. However, they may tend to view emotion generally as something that helps or hinders them. Despite the prevalence of global views about the functionality of emotion overall in the West, there is currently no scale that targets people’s beliefs about emotion overall, independent of their beliefs about whether emotion can be regulated. Specifically, some scales assess people’s beliefs about whether emotions are helpful or harmful in combination with their beliefs about whether emotions can be regulated (Halberstadt et al., 2013) or control behavior and thus cannot be regulated (Leahy, 2002; Veilleux et al., 2015). Other scales focus primarily on beliefs about emotion regulation as well as assessing whether emotions cause harm (Tamir et al., 2007; De Castella et al., 2013). Finally, there are scales that assess the functionality of specific emotions such as feeling upset (Rimes and Chalder, 2010), or specific pleasant and unpleasant feelings (Luong et al., 2015).

Why does the field need another measure? Lay theories about the overall functionality of emotion may affect wellbeing by guiding the strategies people use to regulate emotion. Understanding the antecedents of individual differences in the selection and efficacy of emotion regulation strategies has been noted as an important research direction (Gross, 2015). A great deal of research has examined the consequences of the strategies people use to regulate emotion for their emotion experience, physiology, memory, social interactions, and physical and mental health (e.g., Gross and John, 2003; Ford and Troy, 2019). Relatively little work has addressed what leads people to select particular regulation strategies in the first place. To determine how global beliefs about the functionality of emotion are related to emotion regulation, we need to measure these beliefs in a manner that is not confounded with perceptions of emotion regulation efficacy. It is also important to assess people’s global views about the functionality of emotion overall rather than their views about specific emotional states. These general views should have broad implications for how people respond to emotional events regardless of their specific emotional reaction or how they construe their experience (e.g., as feeling angry, anxious, stressed, or upset). To the extent that emotion is informative and has adaptive functions (e.g., Simon, 1967; Frijda, 1994; Lench et al., 2015), such as guiding goal attainment and providing a primary means of relating to others, the tendency to embrace or avoid one’s emotional life should have important and lasting consequences for people’s wellbeing.

### The Importance of Lay Beliefs About the Overall Functionality of Emotion

Lay theories about the overall functionality of emotion may influence people’s wellbeing in several ways. First, these theories may shape how people appraise and experience emotion. When positive or negative events occur, people who believe that emotion is informative and valuable may be accepting of their emotional responses. Because they do not perceive their feelings to be a threat, they should allow them to unfold more fully and intensely without regretting the experience, instead of ignoring or suppressing their feelings. They may perform well under stress despite experiencing intense emotional and physiological arousal, and recover quickly once distressing events have passed because they do not bear the additional burden of feeling distressed *about* their distress. In contrast, people who view emotion as dysfunctional are likely to feel bad about their emotional reactions. This may prolong unpleasant emotion, make it difficult for people to reason under stress, leading to decreased wellbeing over time.

A second way that lay theories about the overall functionality of emotion may affect wellbeing is by guiding the strategies people use to regulate emotion. Even people who tend to accept their feelings in daily life encounter situations in which they need to regulate or change their emotions to obtain their goals. Why people select one emotion regulation strategy versus another is an under-explored question (Gross, 2015). People’s views about the functionality of emotion may influence the strategies they learn to use and prefer to use (Karnaze and Levine, 2018). People who view emotion as adaptive are likely to accept their emotional reactions to events and attend to them. This would provide them with opportunities to learn when and why they react emotionally including understanding that their emotions reflect their appraisals of events (Frijda, 1988). Understanding the causes of emotions should help people learn to regulate them when necessary by engaging in reappraisal. Viewing emotion as maladaptive would instead motivate people to avoid emotional experiences, mask them, and attempt to get rid of them. This view may promote the use of strategies such as attentional disengagement and distancing, which prevent people from learning from their emotions. This view may also promote more direct attempts to get rid of emotion, such as expressive suppression and substance use, which often have negative consequences (e.g., Gross and Levenson, 1997). Using strategies to avoid, mask, and directly get rid of emotion would prevent people from learning how their appraisals affect their emotional responses, rendering them less effective at engaging in reappraisal.

Finally, people’s theories about the overall functionality of emotion may affect their wellbeing by guiding how they relate to others. People who view emotion as valuable are likely to be more open about and accepting of their own feelings within their relationships. Stress is related to worse relationship satisfaction (Falconier et al., 2015), so people who are less distressed by their own negative emotions should experience more harmonious relationships. People who view emotion as valuable should also be more accepting of how relationship partners feel, and empathy is related to relationship satisfaction (Sened et al., 2017). Both expressing and empathizing with emotion can improve relationship quality and thereby enhance wellbeing (Gross and John, 2003). Those who view emotion as harmful are likely to be less open about their feelings and may also discount or invalidate how relationship partners feel. As a result, they are likely to provide and receive less social support. In summary, we propose that viewing emotion as more helpful than hindering has several benefits, including more effective emotion regulation, promotion of social relationships, and greater wellbeing over time. A primary mechanism underlying these benefits is acceptance of the emotional experiences of the self and others.

To test these ideas, in prior research, we had undergraduates complete a stressful timed reasoning task and questionnaires that assessed their theories of emotion, emotional intensity, emotion regulation strategies, happiness, and social support (Karnaze and Levine, 2018). As a group, participants viewed emotion as more of a help than a hindrance. The more participants endorsed the view that emotion is helpful, the more intense emotion they reported experiencing in daily life, the better they performed on the stressful reasoning task, and the more positive reappraisal, happiness, and social support they reported. In contrast, viewing emotion as a hindrance was associated with reporting greater use of emotion suppression and less social support. Importantly, participants who endorsed a Help Theory about emotion did not do so because their emotional experience was milder. Viewing emotion as helpful was associated with reporting more rather than less intense emotion.

These findings provide preliminary evidence that people’s beliefs about the overall functionality of emotion have consequences for their wellbeing. However, the study had limitations. To assess Help and Hinder Theories, we selected relevant items from existing measures that were not designed to assess beliefs about the overall functionality of emotion and that had differing sets of instructions. As a result, participants may have interpreted some items as referring to positive emotions and others as referring to negative emotions. To capture lay theories about the overall functionality of emotion, a single scale is needed with instructions that encompass both positive and negative emotion. The study was also correlational, thus it was not possible to determine the causal direction of the associations found between lay theories and reasoning, emotion regulation, and wellbeing.

### The Current Investigation

To investigate whether an individual’s theory about the functionality of emotion is a distinct construct with implications for wellbeing and emotion regulation, we conducted two studies. In Study 1, we developed a measure of lay theories about the functionality of emotion, the Help and Hinder Theories about Emotion Measure (HHTEM). The aim was to provide researchers with an efficient means of assessing an individual’s beliefs about the overall functionality of emotion. We assessed the validity and reliability of the measure. We hypothesized that HHTEM scores would show convergent validity by being related in theoretically expected ways with measures of beliefs about emotions and attention to emotions. We hypothesized that HHTEM scores would show discriminant validity by being unrelated, or weakly related, to the need for cognition, approach and avoidance motivation, and social desirability. We assessed criterion correlation, that is, evidence that the HHTEM scores were correlated with relevant measures of emotional experience, emotion regulation, coping strategies, and wellbeing.

In Study 2, we experimentally manipulated the extent to which participants endorsed a Help Theory about emotion. We examined the effect of this manipulation on their emotional and physiological response during and after a distressing film. Previous research has shown that watching films that induce anger, sadness, and disgust increases skin conductance (Kreibig, 2010). Skin conductance is an index of sympathetic nervous system activity and an important component of negative emotion (Dawson et al., 2007). Therefore, we used skin conductance as a measure of physiological arousal. Consistent with our previous finding that more strongly endorsing a Help Theory was correlated with greater self-reported emotional intensity (Karnaze and Levine, 2018), we proposed that encouraging people to view emotion as helpful would lead them to experience more intense emotion as well as greater physiological arousal when viewing distressing events. This greater emotional reactivity would reflect participants’ belief that their emotional reactions are valuable and their willingness to allow those reactions to unfold rather than avoiding or distancing themselves from emotional experiences (Karnaze and Levine, 2018).^1^ Thus, we hypothesized that relative to participants in the control condition, participants who viewed emotion as helpful would: (a) report more intense negative emotion, and exhibit greater sympathetic nervous system activity (SCL), during the distressing film; (b) report greater acceptance of their emotional response and less use of experiential suppression; and (c) show quicker emotional and physiological recovery after the distressing film.

## Study 1

The aim of Study 1 was to create and validate a measure of lay theories about the functionality of emotion, including the fewest items possible, while meeting recommended guidelines of goodness of model-fit indices for confirmatory factor analysis (Acock, 2013). We also assessed the measure’s test–retest reliability and whether scores converged and diverged with scores from other measures in expected ways. Finally, we assessed whether viewing emotion as a help or hindrance was associated with emotion regulation and coping strategies and with wellbeing, to replicate previous findings (Karnaze and Levine, 2018).

### Method

#### Item Development and Pilot Study

We took a systematic approach to conceptualizing and measuring lay theories that emotion helps reasoning and wellbeing and that emotion hinders reasoning and wellbeing. We first consulted functionalist theories of emotion (e.g., Simon, 1967; Schwarz and Clore, 1983; Moors et al., 2013), ethnographic accounts of lay views about emotion (e.g., Lutz, 1986; Parrott, 1995; Shields, 2005), and existing scales assessing lay beliefs about the functionality of specific emotional states or features (Chow and Berenbaum, 2012; Manser et al., 2012; Luong et al., 2015). Based on these accounts, we identified three dimensions along which people commonly view emotion as helpful or as a hindrance. People may view emotion as: (1) motivating/disrupting, (2) informative/irrational, and (3) essential to/a threat to life satisfaction, in ways that do not specifically refer to motivation or rationality. We then generated an over-inclusive pool of items (Loevinger, 1957): six Help items and six Hinder items designed to capture lay beliefs within each of the three dimensions. These 36 initial items were revised based on feedback concerning conceptual clarity and readability from members of the authors’ research team. The complete list of 36 initial items is provided in Supplementary Table 1, which is available online at https://osf.io/4vkfq/https://osf.io/4vkfq/. Because we wanted to assess lay theories that emotion, overall, helps or hinders reasoning and wellbeing, we also developed scale instructions that encouraged participants to think about both positive and negative emotions.

In a pilot study, we administered the initial 36 items to 223 undergraduates at a university in southern California. Participants rated the items in an online questionnaire. To ensure that the final HHTEM included items that were widely interpreted as referring to emotion overall, we had participants answer a follow-up question about each item after they had finished rating all 36 items. For each item, participants indicated whether they had thought mostly about positive emotion, mostly about negative emotion, or about emotion overall, when rating that item. The first step in item selection was to retain the items that more than 40% of participants interpreted as referring to “emotion overall” rather than as referring to mostly positive or mostly negative emotion. This resulted in our retaining 15 items: nine Help Theory, six Hinder Theory.

We conducted an exploratory factor analysis on these 15 items. Two main factors emerged from the data: a factor representing the view that emotion is helpful and a factor representing the view that emotion is a hindrance.^2^ To construct a concise scale, we selected the four items with the highest loadings on a Help Theory factor while including at least one item from each of the three dimensions of a Help Theory. We also selected four items with the highest loadings on a Hinder Theory factor, while including at least one item from each of the three dimensions of a Hinder Theory. The resulting eight-item HHTEM is shown in Appendix A. We then administered and tested the properties of the scale with a separate group of participants.

### Participants

Undergraduates (*N* = 282) at a university in southern California were recruited from the social science subject pool and completed online questionnaires at three time-points for course credit. At each time point, we instructed participants to read each question carefully and complete the questionnaire in a single session. We excluded data from participants who spent less than 10 min on the 90-min questionnaires at Time 1 (*N* = 1) or Time 3 (*N* = 1), or less than 5 min on the 30-min Time 2 questionnaire (*N* = 2). We also excluded data from participants who took more than three standard deviations above the mean time to complete the Time 1 questionnaire (*N* = 7), Time 2 questionnaire (*N* = 2), or Time 3 questionnaire (*N* = 1). The final sample included 282 participants at Time 1 and, due to attrition, 226 participants at Time 2, and 193 participants at Time 3. The mean age of participants was 20.98 years (*SD* = 4.26 years). Reflecting the gender composition of the social science subject pool, 85% of participants were female. Reflecting the ethnic composition of the campus, participants reported their ethnicity as East Asian (45%), Hispanic/Latino (23%), White (18%), Pacific Islander (6%), South Asian (4%), Black (1%), or other (3%).

### Procedure and Measures

Participants completed three online questionnaires. We administered the questionnaires at approximately equal time intervals across the 11-week academic term, avoiding the final 2 weeks of the quarter when students were focusing on final exams. Specifically, they completed the Time 1 questionnaire within the first 7 weeks of the academic term. The questionnaire included the HHTEM and measures used to assess convergent and divergent validity and criterion correlation. Participants completed the Time 2 questionnaire approximately 2 weeks after Time 1 (*M* = 13.94 days, *SD* = 1.74, range = 9–22 days) when the academic term was well underway. This questionnaire assessed participants’ coping strategies as a measure of criterion correlation. Participants completed the HHTEM again as part of the Time 3 questionnaire, approximately 1 month after Time 1 (*M* = 29.63 days, *SD* = 3.21, range = 14–34 days), allowing us to examine test–retest reliability. Preliminary analyses revealed no differences in Help or Hinder Theory endorsement between those who did versus did not complete the Time 2 or Time 3 questionnaires (*p*s > 0.14).

#### Time 1 Questionnaire

The Time 1 questionnaire included the measures listed below.

##### Baseline mood

After a task designed to evoke a neutral affective state (counting trees in photographs of their university), participants rated their current mood using the Positive and Negative Affect Schedule (PANAS; Watson et al., 1988). Baseline positive and negative mood refer to mean ratings of 10 positive (α = 0.92) and 10 negative (α = 0.91) items.

##### HHTEM and convergent measures

Participants then completed the HHTEM. They also completed the following measures of beliefs about the functionality of, and attention given to emotional states, which we expected to be convergent with HHTEM scores. The Affect Valuation scale (Luong et al., 2015) measured how often participants experienced three positive states (joy, contentment, interest) and three negative states (anger, nervousness, downcast) as pleasant, helpful, appropriate, meaningful, and (reverse-coded) as disruptive, unpleasant, inappropriate, and pointless. Ratings were made on a scale from 1 (*almost never or never*) to 7 (*almost always or always*).

The Perceived Affect Utility Scale (Chow and Berenbaum, 2012) assessed how often participants experienced six positive feelings (e.g., proud, appreciative; α = 0.85) and six negative feelings (e.g., fearful, hostile; α = 0.84) as informative, motivational for goal attainment, and beneficial for behavior, using a scale from 1 (*never*) to 6 (*all the time*).

The Following Affective States Test (Gasper and Bramesfeld, 2006) assessed the degree to which participants: attend to and follow their positive feelings (α = 0.75); ignore their positive feelings (α = 0.75); attend to and follow their negative feelings (α = 0.70); and ignore their negative feelings (α = 0.75). Each subscale contained four items, rated from 0 (*strongly disagree*) to 6 (*strongly agree*).

##### Divergent measures

We also assessed measures expected to be divergent. The Short Form of the Need for Cognition Scale (Cacioppo et al., 1996) is an 18-item measure of the tendency to use and enjoy effortful cognition. Participants rated items (e.g., “I would prefer complex to simple problems”) using a scale from 1 (*extremely uncharacteristic*) to 5 (*extremely characteristic*).

BIS/BAS scales (Carver and White, 1994) include four items that assess orientation to approach rewards (behavioral activation system; α = 0.77) and four items that assess orientation to avoid punishment (behavioral inhibition system; α = 0.74), using a scale from 1 (*very true for me*) to 4 (*very false for me*).

The 20-item impression management subscale of the Balanced Inventory of Desirable Responding (Paulhus, 1984) assessed social desirability using a scale from 1 (*not true*) to 7 (*very true*); α = 0.72.

##### Measures of criterion correlation: Emotion regulation, coping strategies, emotional intensity, and wellbeing

The Emotion Regulation Questionnaire (Gross and John, 2003) included five items assessing the use of reappraisal (α = 0.84) and four items assessing the use of expressive suppression (α = 0.68), using a scale from 1 (*strongly disagree*) to 7 (*strongly agree*).

Participants also completed the Brief COPE Inventory (Carver, 1997) which assessed how often participants used different coping strategies when experiencing stress, including two items each for: active coping (α = 0.66), planning (α = 0.68), positive reframing (α = 0.78), acceptance (α = 0.71), receiving emotional support (α = 0.89), seeking instrumental support from others (α = 0.86), and substance use (α = 0.94). The scale ranged from 1 (*I usually don’t do this at all*) to 4 (*I usually do this a lot*).

The six-item Impulse Strength factor of the Berkeley Expressivity Questionnaire (Gross and John, 1995) assessed the intensity of participants’ emotional reactions, using a scale from 1 (*strongly disagree*) to 7 (*strongly agree*); α = 0.86.

Finally, participants completed four measures of wellbeing. The Satisfaction with Life Scale (Diener et al., 1985) included five statements about satisfaction with life, rated from 1 (*strongly disagree*) to 7 (s*trongly agree*); α = 0.87.

The four-item Subjective Happiness Scale (Lyubomirsky and Lepper, 1999) assessed participants’ level of general happiness by asking participants to compare themselves to happy and unhappy individuals on a 7-point scale (α = 0.84).

The 12-item Multidimensional Scale of Perceived Social Support (Zimet et al., 1988) assessed feelings of support by family, friends, and a significant other, using a scale from 1 (*strongly disagree*) to 5 (*strongly agree*); α = 0.94.

The 10-item version of the Center for Epidemiologic Studies Depression Scale (Andresen et al., 1994) assessed how often participants felt symptoms during the past week (e.g., “I could not ‘get going”’) using a scale from 1, (*rarely or none of the time/less than 1 day*) to 4 (*all of the time/5–7 days*); α = 0.85.

#### Time 2 Questionnaire

To further assess criterion correlation in the midst of the academic term, participants again completed the emotion regulation and coping measures that they had completed at Time 1.

#### Time 3 Questionnaire

At Time 3, to assess test-retest reliability, participants again completed the HHTEM as well as the convergent measures described above for Time 1.^3^

### Results

#### Psychometric Properties of the HHTEM

Table 1 presents descriptive data on the HHTEM. Preliminary analyses showed no significant differences between genders or ethnic groups in their endorsement of help or hinder theories (all *p*s > 0.22). As found by Karnaze and Levine (2018), participants tended to view emotion overall as more helpful (*M* = 3.43, *SD* = 0.62) than hindering (*M* = 3.11, *SD* = 0.62), *t*(280) = 6.25, *p* < 0.001. Cronbach’s alpha was 0.74 for Help items and 0.64 for Hinder items. Average item intercorrelations were 0.42 for Help items and 0.32 for Hinder items. Responses on both scales followed a normal distribution. Help Theory and Hinder Theory endorsement were not correlated with one another, *r*(280) = −0.13, *p* = 0.83.

**TABLE 1:** Descriptive data for the Help and Hinder Theories about Emotion Measure in Study 1.

**Psychometric property**	**Help Theory**	**Hinder Theory**
*N*	280	280
*M*	3.43	3.11
*SD*	0.62	0.62
Range	1.25 – 5.00	1.00 – 5.00
Kurtosis	0.73	0.87
Skewness	−0.07	0.23
Cronbach’s α	0.74	0.64
Mean item intercorrelation	0.42	0.32

##### Factor structure

**FIGURE 1:**
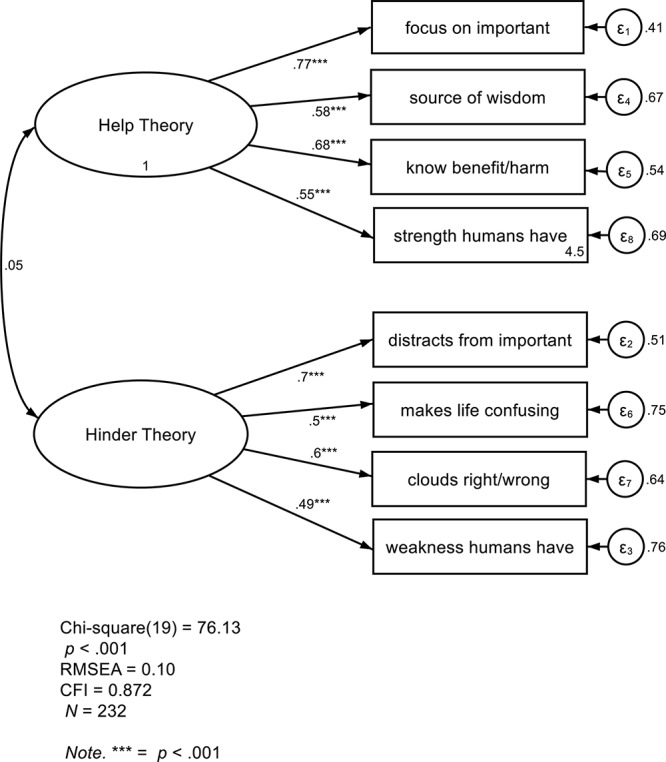
Study 1 confirmatory factor analysis with the distinct Help Theory and Hinder Theory factors, with standardized regression coefficients displayed.

Figure 1 shows the results of a confirmatory factor analysis that modeled Help and Hinder Theories at Time 1 as distinct factors which were allowed to covary. We also followed the approach of Judd et al. (1986) to test the hypothesis that Help and Hinder items were better represented as measuring two distinct constructs rather than one bipolar construct. In the first step of this process, Model 1 tested whether the four Help Theory items and the four Hinder Theory items could be represented as one bipolar Help-Hinder Theory latent factor. Model 1 did not show a good fit to the data; RMSEA = 0.19; CFI = 0.543. The absolute values of standardized loadings of the eight items ranged from 0.05 to 0.75. Model 2 then tested whether Help Theory items loaded significantly onto a latent factor of Help Theory, and whether Hinder Theory items loaded significantly onto a latent factor of Hinder Theory, with these factors allowed to covary. This model showed a better fit to the data, however CFI and RMSEA did not meet recommended guidelines (Acock, 2013); RMSEA = 0.10; CFI = 0.872. The standardized loadings of the four Help Theory items ranged from 0.58 to 0.77. The standardized loadings of the four Hinder Theory items ranged from 0.49 to 0.70.

To determine whether Model 2 was a significantly better fit than Model 1, we conducted a *X*^2^ test comparing the fit of the two models. The two-factor model was a statistically significant improvement over the one-factor model, *X*^2^(1) = 148.24, *p* < 0.001. Because Help Theory and Hinder Theory were not related, we also represented Help Theory and Hinder Theory in separate models. In the Help Theory model, CFI (0.996) and RMSEA (0.04) both met the criteria considered for a good fit to the data (CFI ≥ 0.95; RMSEA ≤ 0.05). In the Hinder Theory model, CFI (0.973) met the criteria for a good fit to the data, but RMSEA (0.08) did not meet the recommended cutoff. In summary, fit indices were better when modeling Help and Hinder Theories as separate factors rather than as one Help-versus-Hinder Theory factor. Separate models of Help and Hinder Theories had model-fit indices that exceeded the recommended criteria for a good fit (CFI ≥ 0.95; RMSEA ≤ 0.05).

##### Test–retest reliability

For convergent measures, test–retest reliability coefficients ranged from 0.28 to 0.85 (e.g., 0.28 and 0.85 for valuation of negative and positive emotion, respectively, and 0.65 and 0.57 for utility of positive and negative emotion), and are available online in Supplementary Table 4. Test–retest reliability for Help and Hinder scores fell at about the middle of this range. Participants’ Help Theory scores were correlated between Time 1 and Time 2, *r*(280) = 0.46, *p* < 0.001. Hinder Theory scores were also correlated between Time 1 and Time 2, *r*(199) = 0.50, *p* < 0.001.

### Convergent and Discriminant Validity and Criterion Correlation

We conducted correlation analyses between the HHTEM subscales and validity measures using bootstrapping. This involved taking 1,000 random samples from the data, with replacement, to compute an interval for each correlation coefficient (e.g., the correlation between Help Theory and Perceived Affect Utility scores) to obtain a 95% probability that the interval contains the correlation coefficient of the population. If an interval does not include zero, the association can be interpreted as statistically significant. The results are shown in Table 2. We describe the results below using the conventional descriptions of correlations as weak (*r* < 0.20), moderate (0.20 ≥ *r* ≥ 0.50), and strong (*r* > 0.50; Hemphill, 2003). If either Help or Hinder Theory endorsement was correlated with a variable, but the other Theory was not, the Table also presents a *z*-test indicating whether the strength of the correlation differed significantly for Help versus Hinder Theory (Steiger, 1980). See Supplementary Tables 5–8 for correlations among convergent measures, among divergent measures, and among emotion regulation measures.

**TABLE 2:** Study 1 correlations of HHTEM subscales with convergent and divergent measures and tests of the difference between dependent correlations.

	**Help Theory**		**Hinder Theory**		
**Measure**	***r***	**95% CI**	***r***	**95% CI**	***z***
Convergent measures					
Value of Specific Affective States					
Positive affect valuation^a^	0.20**	[0.06, 0.33]	–0.11	[−0.23, 0.01]	3.70***
Negative affect valuation^a^	0.05	[−0.11, 0.19]	–0.01	[−0.14, 0.12]	–
Positive affect utility^b^	0.25***	[0.13, 0.36]	–0.01	[−0.14, 0.12]	3.12**
Negative affect utility^b^	0.15*	[0.03, 0.27]	–0.14	[0.00, 0.26]	–
Attention to Specific Affective States^c^					
Attention to positive feelings	0.29***	[0.16, 0.40]	–0.01	[−0.14, 0.12]	3.63***
Attention to negative feelings	0.17**	[0.05, 0.30]	0.20**	[0.07, 0.31]	–
Ignoring positive feelings	−0.23**	[−0.29, −0.01]	−0.23**	[0.09, 0.35]	–
Ignoring negative feelings	−0.17*	[−0.30, −0.06]	−0.17**	[0.22, 0.49]	–
Divergent measures					
Need for Cognition	0.06	[−0.05, 0.17]	–0.07	[−0.18, 0.05]	–
Approach motivation	0.01	[−0.14, 0.15]	0.06	[−0.09, 0.20]	–
Avoidance motivation	0.04	[−0.10, 0.18]	0.01	[−0.13, 0.14]	–
Social desirability	–0.12	[−0.23, −0.01]	–0.05	[−17, 0.07]	–

^*a*^Affect Valuation scale (Luong et al., 2015); ^*b*^Perceived Affect Utility Scale (Chow and Berenbaum, 2012); ^*c*^Following Affective States Test (Gasper and Bramesfeld, 2006). Participants completed all measures at Time 1. *Z-*test values are presented for all measures that were significantly correlated only with Help Theory or only with Hinder Theory. *p < 0.05. **p < 0.01. ***p < 0.001.

#### Convergent Measures for Help and Hinder Theories

We first assessed whether Help and Hinder Theory endorsement were related to existing measures of beliefs about the functionality of specific emotional states or features.

##### Valuation of positive and negative feelings

As Table 2 shows, when examining associations with the Affect Valuation Scale, we found that Help Theory endorsement was weakly correlated with valuing positive feelings but was not correlated with valuing negative feelings (see Supplementary Table 5 for correlations among convergent measures). Hinder Theory endorsement was not correlated with valuing positive or negative feelings. The Affect Valuation Scale includes questions about how appropriate and how enjoyable it is to experience positive and negative feelings. Therefore, we conducted follow-up analyses in which we examined correlations between Help and Hinder Theory endorsement and scale items that specifically assessed how meaningful, helpful, pointless, or disruptive feelings are. As expected, Help Theory endorsement was correlated with viewing both positive feelings, *r*(280) = 0.30, *p* < 0.001, and negative feelings as meaningful, *r*(280) = 0.23, *p* < 0.001, and with viewing both positive feelings, *r*(280) = 0.31, *p* < 0.001, and negative feelings as helpful, *r*(280) = 0.19, *p* = 0.003. As expected, Hinder Theory endorsement was correlated with viewing both positive feelings, *r*(280) = 0.17, *p* = 0.004, and negative feelings as pointless, *r*(280) = 0.14, *p* = 0.01, and with viewing positive feelings as disruptive, *r*(280) = 0.18, *p* = 0.002.

##### Perceived utility of positive and negative feelings

Table 2 shows that, as expected, Help Theory endorsement was moderately correlated with viewing both positive and negative feelings as useful on the Perceived Affect Utility Scale (Chow and Berenbaum, 2012). Hinder Theory was not correlated with viewing either positive or negative feelings as useful.

##### Attention to positive and negative feelings

Examining responses to the Following Affective States Test (Gasper and Bramesfeld, 2006), Help Theory endorsement was moderately correlated with paying attention to and following positive feelings, and was weakly correlated with attending to and following negative feelings. In contrast, Hinder Theory endorsement was moderately correlated with ignoring both positive and negative feelings.

#### Divergent Measures for Help and Hinder Theories

We next assessed whether Help and Hinder Theory endorsement were unrelated or weakly related to constructs that should be theoretically distinct from viewing emotion as a help or hindrance.

##### Need for cognition

As expected, neither Help nor Hinder Theory was related to need for cognition. Thus, viewing emotion as helpful did not reflect valuing cognition less. Viewing emotion as a hindrance did not reflect valuing cognition more.

##### Motivation

Neither Help nor Hinder Theory endorsement was related to approach or avoidance motivation. Thus, Help Theory endorsement did not reflect a tendency to approach rewarding experiences, which would increase positive feelings. Hinder Theory endorsement did not reflect a tendency to avoid negative experiences, which would decrease negative feelings.

##### Social desirability

As expected, Help and Hinder Theory endorsement were not related to the tendency to present oneself in socially desirable ways.

#### Criterion Correlation

To assess the criterion correlation of the HHTEM, we examined how Help and Hinder Theory endorsement were related to: (a) emotion regulation and coping strategies (assessed at both Time 1 and Time 2), (b) emotional experience (Time 1), and (c) measures of wellbeing (Time 1).

##### Emotion regulation and coping strategies

The results for emotion regulation and coping strategies are shown in Table 3. Consistent with our past research (Karnaze and Levine, 2018), Help Theory endorsement was weakly correlated with engaging in reappraisal both relatively early in the academic term (Time 1) and in the midst of the academic term (Time 2). Help Theory endorsement was also weakly associated with acceptance at Time 2, and with the use of planning to cope with stress at both time points and positive reframing at Time 1. Help Theory endorsement was moderately correlated with seeking and receiving social support at both time points. In contrast, Hinder Theory endorsement was weakly correlated with using expressive suppression to regulate emotion at Time 1, and weakly-to-moderately correlated with using substances to cope at both time points. As Table 3 shows, although there were a few exceptions, most associations found between HHTEM scores and emotion regulation and coping strategies were consistent over time.

**TABLE 3:** Study 1 correlations of HHTEM subscales with emotion regulation and coping strategies at Time 1 and Time 2, and tests of the difference between dependent correlations.

	**Time 1 questionnaire**					**Time 2 questionnaire**				
	**Help Theory**		**Hinder Theory**		**Difference**	**Help Theory**		**Hinder Theory**		**Difference**
Measure	**r**	**95% CI**	**r**	**95% CI**	**z**	**r**	**95% CI**	**r**	**95% CI**	**z**
Emotion regulation										
Reappraisal	0.18**	[0.04,0.31]	0.01	[−0.11, 0.11]	2.20*	0.18**	[0.01, 0.33]	0.01	[−0.12, 0.14]	1.37
Expressive suppression	–0.01	[−0.13, 0.13]	0.17**	[0.04, 0.31]	−2.13*	–0.01	[−0.16, 0.14]	0.08	[−0.05, 0.23]	–
Coping strategies										
Acceptance	0.08	[−0.05, 0.22]	–0.01	[−0.13, 0.10]	–	0.17*	[0.02, 0.29]	0.07	[−0.05, 0.19]	1.04
Active coping	0.18	[0.06, 0.32]	0.01	[−0.13, 0.13]	–	0.13	[−0.01, 0.25]	–0.02	[−0.17, 0.12]	–
Planning	0.18**	[0.05, 0.31]	0.03	[−0.12, 0.12]	1.78	0.16*	[0.01, 0.31]	0.07	[−0.08, 0.20]	0.93
Positive reframing	0.16**	[0.02, 0.29]	0.03	[−0.08, 0.15]	1.54	0.15	[0.01, 0.29]	0.03	[−0.09, 0.14]	–
Instrumental social support	0.21***	[0.08, 0.34]	0.02	[−0.09, 0.14]	2.26*	0.21**	[0.07, 0.37]	0.03	[−0.10, 0.16]	1.87
Emotional social support	0.22***	[0.10, 0.34]	0.10	[−0.03, 0.22]	1.44	0.28***	[0.15, 0.41]	0.02*	[−0.10, 0.14]	2.74**
Substance use	0.12	[0.00, 0.24]	0.25***	[0.14, 0.35]	–1.57	–0.02	[−0.16, 0.13]	0.18**	[0.05, 0.30]	−2.07*

Participants completed the HHTEM at Time 1. They completed the Emotion Regulation Questionnaire (Gross and John, 2003) and the Brief COPE Inventory (Carver, 1997) at both Time 1 and Time 2. *Z-*test values are presented for all measures that were significantly correlated only with Help Theory or only with Hinder Theory *p < 0.05. **p < 0.01. ***p < 0.001.

##### Emotional experience

Participants rated their baseline positive and negative mood after completing a neutral filler task at the start of the Time 1 questionnaire. Help Theory endorsement was weakly correlated with a more positive baseline mood but was not correlated with negative baseline mood. In contrast, Hinder Theory endorsement was moderately correlated with a more negative baseline mood but was not correlated with positive baseline mood. Help Theory endorsement was moderately correlated with greater emotional intensity on the Impulse Strength factor of the Berkeley Expressivity Questionnaire (Gross and John, 1995), whereas Hinder Theory was not related to emotional intensity.

##### Wellbeing

We hypothesized that, even after adjusting for differences in baseline mood, participants who more strongly endorsed a Help Theory would report more happiness, life satisfaction, and social support, and fewer depressive symptoms. We expected participants who more strongly endorsed a Hinder Theory to report less happiness, life satisfaction, and social support, and more depressive symptoms. To test this, we conducted separate hierarchical regression analyses for each outcome. In each analysis, we entered baseline positive and negative mood at Step 1, and entered Help and Hinder Theory endorsement in Step 2.

As shown in Table 4, positive and negative baseline mood showed the expected associations with each outcome variable. That is, a more positive mood at baseline was associated with greater happiness, life satisfaction, and social support, and fewer depressive symptoms. A more negative mood at baseline was associated with less happiness, life satisfaction, and social support, and more depressive symptoms. At Step 2, after accounting differences in baseline mood, participants who more strongly endorsed a Help Theory reported more social support. They also showed a non-significant tendency to report more satisfaction with life (*p* = 0.052). Participants who more strongly endorsed a Hinder Theory reported less happiness and more depressive symptoms. These findings are consistent with our hypothesis that endorsing a Help Theory would be associated with greater wellbeing and that endorsing a hinder would be associated with less wellbeing.

**TABLE 4:** Study 1 summary of hierarchical regression analyses for Help and Hinder Theory endorsement predicting wellbeing outcomes (*N* = 282).

	**Happiness**		**Life satisfaction**		**Social support**		**Depressive symptoms**	
**Variable**	**Δ*R*^2^**	**β**	**Δ*R*^2^**	**β**	**Δ*R*^2^**	**β**	**Δ*R*^2^**	**β**
Step 1	0.18***		0.19***		0.09***		0.30***	
Baseline positive mood		0.35***		0.36***		0.23***		−0.23***
Baseline negative mood		−0.31***		−0.30***		−0.23***		0.53***
Step 2	0.02*		0.02*		0.05**		0.05**	
Baseline positive mood		0.35***		0.35***		0.19**		−0.25***
Baseline negative mood		−0.28***		−0.29***		−0.24***		0.46***
Help Theory		0.05		0.11^†^		0.21***		0.11*
Hinder Theory		−0.13*		–0.08		–0.06		0.21***
Total *R*^2^	0.45***		0.20***		0.37***		0.59***	

^†^*p* = 0.05. *p < 0.05. **p < 0.01. ***p < 0.001.

Contrary to our hypotheses, participants who more strongly endorsed a Help Theory also reported more depressive symptoms. People who view their emotions as helpful may consider their depressive symptoms, which included both emotions and behaviors, as important and thus attend to them and even share them with others, which could inadvertently prolong the duration of depressive symptoms. This is supported by the finding that emotional experiences are prolonged when people continue to think about them, or share them with others (Verduyn et al., 2011). However, to further explore this unexpected finding, we computed a dichotomous variable representing whether participants did (coded as 1; 64%) or did not (coded as 0) meet the cutoff level of ≥10 for clinically significant depressive symptoms (e.g., Thielke et al., 2010). We then computed partial correlations, controlling for baseline positive and negative emotion, between Help and Hinder Theories and this dichotomous variable. The results showed that endorsing a Hinder Theory, *r_*partial*_* = 0.13, *p* < 0.05, but not a Help Theory (*p* = 0.18), was associated with clinically significant depressive symptoms.

### Discussion

In Study 1, we created a new measure of lay theories about the functionality of emotion. Across two samples of university students (the pilot study and Study 1 samples), we demonstrated that the model representing Help and Hinder Theories about emotion as distinct constructs was a better fit to the data than modeling them as a unipolar construct. The alpha coefficients, a measure of scale reliability, were on the lower side of the range considered to be acceptable, particularly for the hinder scale. This was likely due to including only four, relatively heterogeneous, items. We sought to create a measure that was short and easy to administer while including unique rather than redundant items in order to capture the broad constructs of viewing emotion overall as helpful or hindering for reasoning, goal-pursuit, and general wellbeing. We expected a scale that measured these broad lay theories to predict a range of outcomes and behaviors. Having a narrow range of items may produce indices of high internal consistency (e.g., alpha), but be less useful for predicting outcomes of interest. Likewise, having too much heterogeneity can result in less accuracy in predicting outcomes. This is known as the bandwidth-fidelity tradeoff (Cronbach and Gleser, 1957). Additional Hinder Theory items may increase Cronbach’s alpha. However, because alpha is a function of scale length and item homogeneity, John and Soto (2007) recommend also computing the average item intercorrelations. These values for both Help Theory (0.42) and Hinder Theory (0.32) suggest that the items within each construct are moderately related.

The test–retest reliability coefficients for Help theory (0.48) and Hinder Theory (0.51) were low. It should be noted that the test-retest reliability coefficients for related measures in the literature (0.28 for valuing negative feelings, 0.57 for perceiving negative feelings as useful) were also low (Supplementary Table 4). We also found that the test-retest reliability coefficients for the scales assessing attention to positive and negative feelings ranged from 0.56 to 0.67, and in previous research, they ranged from 0.59 to 0.67 (Gasper and Bramesfeld, 2006). Taken together, these findings suggest that beliefs about emotional experiences are less stable over time than other constructs such as beliefs about cognition, attitudes, or personality traits. Scores for scales assessing beliefs about emotion should rely on memory for past emotional experiences, which can be shaped factors such as current feelings or current appraisals of past emotional experiences (Levine et al., 2018). Scale modification in further research should encourage participants to think about emotions more generally, rather than in relation to their current circumstances.

Scores for the Help Theory subscale of the HHTEM reflect the extent to which people view both positive and negative emotions as meaningful and helpful. Hinder Theory endorsement reflects the extent to which participants view both positive and negative emotions as pointless and view positive emotions as disruptive. Help Theory endorsement does not simply reflect the tendency to pursue goals to obtain rewards, nor does it reflect less need for cognition. Hinder Theory endorsement does not simply reflect the tendency to ignore feelings, differences in emotional intensity, or greater need for cognition. Thus, both Help and Hinder Theory scores were related to, and distinct from, other measures in theoretically expected ways, providing evidence for convergent and discriminant validity.

Consistent with our prior findings (Karnaze and Levine, 2018), Help and Hinder Theory scores also predicted emotion regulation, coping strategies, and wellbeing in theoretically expected ways, providing evidence of criterion correlation. Specifically, viewing emotion overall as a hindrance was associated with using expressive suppression (at Time 1) and substances to regulate emotions, experiencing less happiness, and with a greater likelihood of experiencing clinically significant depressive symptoms. Viewing emotion overall as helpful was associated with emotional acceptance (at Time 2), with using reappraisal to regulate emotion, and with reporting more social support. In addition, a non-significant tendency was found for participants who viewed emotion as helpful to report more life satisfaction (*p* = 0.052). One way that Help Theory may confer wellbeing is by promoting acceptance of emotional experience, which in turn allows people to recover quickly from distressing events. However, the data in Study 1 were correlational, leaving uncertainty about the causal direction of the associations. To test the hypothesis that endorsing a Help Theory about emotion promotes greater acceptance of emotion during distressing events, and reduced emotional and physiological reactivity after distressing events, we manipulated people’s beliefs about the functionality of emotion in Study 2.

## Study 2

Study 2 assessed whether viewing emotion as helpful influenced people’s emotional and physiological response, and regulatory strategies, when faced with distressing events. Specifically, we manipulated participants’ views about the value of emotion and assessed the effects on their emotional response, physiological reactivity, and regulatory strategies during a distressing film and their recovery after the film. In Study 1, the more people viewed emotion overall as helpful, the more they reported using acceptance to cope with stressful experiences. We also found that people who viewed emotion overall as helpful reported experiencing more intense emotional reactions (Karnaze and Levine, 2018). People who are led to view emotion as helpful should be more accepting of their emotional responses to distressing events and thus more fully experience them, resulting in more intense emotional and physiological reactions during such events. After distressing events have passed, however, people who accept their emotions should feel less distressed *about* their reactions, resulting in less distress overall. Therefore, we hypothesized that, during a distressing film, participants encouraged to view emotion as helpful, compared to those in the control condition, would report more intense negative emotion, exhibit greater sympathetic nervous system activity (skin conductance level; SCL), and report more acceptance and less experiential suppression of emotion. We hypothesized that after the distressing film, participants encouraged to view emotion as helpful would show faster recovery.^4^

### Method

#### Participants

Undergraduates (*N* = 160) were recruited from the social sciences subject pool and via flyers at a university in southern California for a study on responses to multimedia. Participants were compensated with course credit (subject pool) or $10 (flyers). A power analysis of previous studies assessing emotion regulation and skin conductance responses to film clips, conducted with the program G^∗^Power, showed that 120 participants (60 per condition) were required to obtain a power of 0.80. The experimental manipulation required students to provide open-ended responses describing how emotion was helpful. We excluded data from seven participants who did not complete these two questions. We excluded data from four other participants due to experimenter or program error. The mean age of participants was 20.55 years (*SD* = 2.61) and most (80%) were female. Participants were Asian (*n* = 43%), Hispanic/Latino (29%), White (15%), African American (2%), Mixed Race (5%), or other race-ethnicity (6%).

#### Procedure and Measures

Participants sat in a corner with two adjacent computer desks. They rotated the chair between the computer monitors at these two desks during the session. Film clips and questions during the post-film period were administered via a computer set up with E-Prime^®^ 2.0 software that allowed start and stop times to be marked in the physiological data. The other study materials were administered on a computer with a Qualtrics questionnaire.

##### Physiological measures

At the beginning of the session, the investigator attached two silver–silver chloride electrodes to the palm of the hand that participants did not use for the computer mouse and fitted each participant with a respiratory transducer snugly over their clothes. Skin conductance and respiration were measured continuously. We used an E-Prime program to send time markers to the electrodermal activity (EDA) data file at the beginning and end of three events. This allowed us to compute average SCL for each event: (a) a 2.5-min neutral film, (b) a 4-min distressing film, and (c) post-film completion of retrospective ratings of emotions and regulation strategies used during the distressing film. EDA data were processed with BioLab Acquisition Software and any EDA changes associated with sudden changes in respiration were transformed using the spline interpolation function in the EDA Analysis 3.1.2 program.

##### Neutral film and baseline SCL and emotion

To assess baseline SCL, participants watched a film clip of nature scenes that has been recommended for inducing a neutral mood (Rottenberg et al., 2007). Participants rated the greatest amount of positive emotion (compassion, happiness, interest, pride; α = 0.78) and negative emotion (anger, anxiety, confusion, contempt, disgust, embarrassment, fear, guilt, sadness, shame, unhappiness; α = 0.80) they felt during the film, using a scale from 1 (*not at all/none*) to 9 (*extremely/a great deal*).

##### Experimental manipulation

Participants were randomly assigned to a Help Theory condition or control condition. After watching the neutral film and rating their emotions, participants assigned to the Help Theory condition read and summarized brief article excerpts. The excerpts cited purported scientific evidence that experiencing emotion helps the pursuit of goals, physical health, mental health, and relationship satisfaction. Participants then wrote about how their own personal experiences of pleasant and unpleasant emotions were helpful in their transition to life as a college student. Finally, participants were asked to give advice about how emotion is helpful to an incoming college freshman, Taylor, who was assigned to live in a triple dormitory room in the upcoming year. Participants who were assigned to the control condition read and summarized brief article excerpts citing purported scientific evidence that verbal ability is helpful to pursuing goals, physical health, mental health, and relationship satisfaction. Participants were prompted to write about how their own personal experiences of how oral and written communication were helpful to their transition to life as a college student. They gave advice about how verbal ability is helpful, to an incoming college freshman, Taylor, who was assigned to live in a triple dormitory room in the upcoming year.

##### Hinder Theories about Emotion Measure

After the manipulation, participants were instructed, “Earlier, you read some passages about whether [emotion/verbal ability] is helpful or harmful. Your personal experience might lead you to agree or disagree with what you read. Next, we are interested in your own personal views about the extent to which emotion is helpful or harmful. We want to know what you think, rather than what the experts think.” Participants then completed the 8-item HHTEM.

##### Distressing film

Participants then watched a 4-min excerpt from the film *Cry Freedom*, which depicts soldiers shooting and killing schoolchildren in South Africa. This film clip has been shown to elicit a range of negative emotions, and to provide an ecologically valid way to assess how people react emotionally to distressing events and regulate negative emotion (Rottenberg et al., 2007).

##### Emotional response and regulation strategies

Immediately after the film, participants rated the greatest intensity of their positive (α = 0.48) and negative emotional responses (α = 0.76) to the distressing film using the same questions and scales used for the neutral film. They also retrospectively reported the strategies they had used to regulate their emotional response during the film. Using items adapted from Tull et al. (2010), participants rated how much they had engaged in acceptance (“I let myself feel whatever I was feeling”) and reappraisal (“I tried to think differently about the events in order to change how I was feeling about the film”), using a scale from 1 (*not at all*) to 9 (*very much*). They also rated the extent to which they used experiential suppression (“I tried not to feel how I was feeling” and “I tried to stop my emotions”) using the same scale. They also answered the question, “How much did watching the film affect your mood during the film?” using a scale from 1 (*not at all*) to 9 (*a great deal*).

##### Post-film period SCL

Because SCL reactivity can decrease quickly, to assess physiological recovery, we examined SCL in the post-film period, during which participants retrospectively rated how they felt and regulated emotion during the distressing film.

##### Emotion and emotion regulation during the four-minute rest period

We then told participants to rest for a few minutes while the recording program recalibrated. The program advanced to the next set of questions after 4 min. Participants were asked to rate the degree to which they felt positive (α = 0.62) and negative emotions (α = 0.93) during the 4-min rest period. They also rated the extent to which they used the emotion regulation strategies during the rest period. Participants also rated, “How much did watching the last film affect your current mood?” using a scale from 1 (*not at all*) to 9 (*a great deal*).

##### Violence rating and prior exposure to the film

Participants rated how violent the film was compared to what they watch in a typical week, from 1 (*much less violent*) to 7 (*much more violent*). They also indicated whether they had seen the film before and how much they knew about the events in the film.

##### Debriefing

Finally, participants completed demographic questions and watched an amusing film clip to induce a more positive mood. During debriefing, participants were told that we created the article excerpts for the study, and that research suggests that emotional experience can have both positive and negative consequences.

### Results

#### Preliminary Analyses

We compared HHTEM ratings across conditions to find out whether we successfully manipulated the extent to which participants viewed emotion as helpful. Participants in the Help Theory condition endorsed a Help Theory (*M* = 3.74, *SD* = 0.64) more than did those in the control condition (*M* = 3.40, *SD* = 0.65), *t*(158) = 3.22, *p* < 0.01, *d* = 0.09. Participants in the Help Theory condition endorsed a Hinder Theory (*M* = 2.95, *SD* = 0.60) less than those in the control condition (*M* = 3.17, *SD* = 0.61), *t*(158) = −2.33, *p* < 0.05, *d* = 0.07. Thus, the experimental manipulation was successful. Alpha values were 0.73 for Help Theory items and 0.58 for Hinder Theory items. The mean item intercorrelations were 0.40 for Help Theory items and 0.25 for Hinder Theory items. Preliminary analyses showed no difference between the Help Theory condition (*M* = 1.70, *SD* = 0.84) and the control condition (*M* = 1.80, *SD* = 0.91) in negative affect during the neutral film *t*(151) = 0.74, *p* = 0.46, *d* = 0.11. There was no difference between conditions in how violent they found the distressing film compared to what they watch in a typical week (*p* = 0.21).

#### Emotional Response to the Distressing Film

To assess whether endorsing a Help Theory affected participants’ subjective emotional response to the distressing film, we conducted a 2 (Help Theory vs. control condition) × 3 (time: neutral film, distressing film, 4-min rest period) mixed model ANOVA on mean negative emotion. Only a main effect of time was found. Negative emotion increased from the neutral film (*M* = 1.76, *SD* = 0.88), to the distressing film (*M* = 6.10, *SD* = 1.30), and decreased during the post-film rest period (*M* = 2.82, *SD* = 1.75), *F*(2,302) = 614.23, *p* < 0.001, η^2^ = 0.80. Negative emotion did not differ by condition, *F*(1,151) = 1.07, *p* = 0.30, nor was there an interaction between time and condition, *F*(1,151) = 0.26, *p* = 0.61, η^2^ = 0.01.

We also conducted a 2 (condition) × 2 (time: distressing film, 4-min rest period) mixed model ANOVA on the extent to which participants reported that the distressing film affected their mood. A main effect of time indicated that, overall, participants’ current mood was affected more during the distressing film than afterward, *F*(1,151) = 110.67, *p* < 0.001, η^2^ = 0.42. An interaction between time and condition was also found, *F*(1,150) = 5.98, *p* = 0.02, η^2^ = 0.04. The extent to which the distressing film affected participants mood during the film did not differ significantly for participants in the Help Theory condition (*M* = 7.12, *SD* = 1.82) and the control condition (*M* = 7.12, *SD* = 2.01), *t*(151) = 0.01, *p* = 0.99. After the distressing film, however, participants in the Help Theory condition reported that their mood was less affected (*M* = 5.07, *SD* = 2.55) than those in the control condition (*M* = 5.85, *SD* = 2.11), *t*(151) = 2.06, *p* = 0.04, *d* = 0.34. Thus, inducing a Help Theory did not affect the intensity of specific negative emotions reported during or after the distressing film. After the film, however, participants in the Help Theory condition reported that the film was affecting their current mood less than those in the control condition.^5^

#### Physiological Response to the Distressing Film

Preliminary analyses of physiological reactivity showed that, overall, the higher participants’ SCL during the neutral film, the higher their SCL during the distressing film, *r*(156) = 0.88, *p* < 0.001. In addition, the more violent participants rated the distressing film compared to films they typically watched, the higher their SCL during the distressing film, controlling for neutral film SCL, *r*_*partial*_(154) = 0.17, *p* = 0.03. Therefore, we adjusted for neutral film SCL and violence rating in analyses comparing SCL between conditions during or after the distressing film. In analyses that included SCL during the neutral film, we adjusted for violence rating.

To find out if physiological reactivity differed between conditions, we first conducted a 3 (time: neutral film, distressing film, post-film) × 2 (condition) mixed model ANCOVA on mean SCL, with violence rating as the covariate. The results showed an interaction between time and condition, *F*(1,154) = 5.72, *p* = 0.02, η^2^ = 0.04. We then compared SCL in the Help Theory and control conditions separately at each time point. During the neutral film, SCL did not differ between the Help Theory condition (*M_*adjusted*_* = 6.70, *SD* = 5.25) and control condition (*M*_*adjusted*_ = 5.86, *SD* = 4.38), *F*(1,154) = 1.21, *p* = 0.27, η^2^ = 0.01. As hypothesized, during the distressing film, participants in the help condition showed higher SCL (*M*_*adjusted*_ = 8.07, *SD* = 5.35) than participants in the control condition (*M*_*adjusted*_ = 7.27, *SD* = 4.71), *F*(1,153) = 4.37, *p* = 0.04, η^2^ = 0.03. After the distressing film, SCL did not differ between the Help Theory condition (*M*_*adjusted*_ = 7.54, *SD* = 5.42) and control condition (*M*_*adjusted*_ = 7.23, *SD* = 4.69), *F*(1,153) = 0.51, *p* = 0.48, η^2^ = 0.01.

To test our *a priori* hypothesis that viewing emotion as helpful would promote physiological recovery, we also compared mean SCL (unadjusted) during versus after the distressing film separately for each condition. SCL decreased significantly after the distressing film for participants in the Help Theory condition, *t*_*paired*_(68) = 2.50, *p* = 0.02, *d* = 0.11, but not for participants in the control condition *t*_*paired*_(87) = −0.042, *p* = 0.97, *d* = 0.01. In summary, participants who viewed emotion as helpful showed greater physiological reactivity during the distressing film but showed recovery after the film. After the distressing film, participants in the Help Theory condition did not differ in reactivity from those in the control condition.

#### Emotion Regulation

**FIGURE 2:**
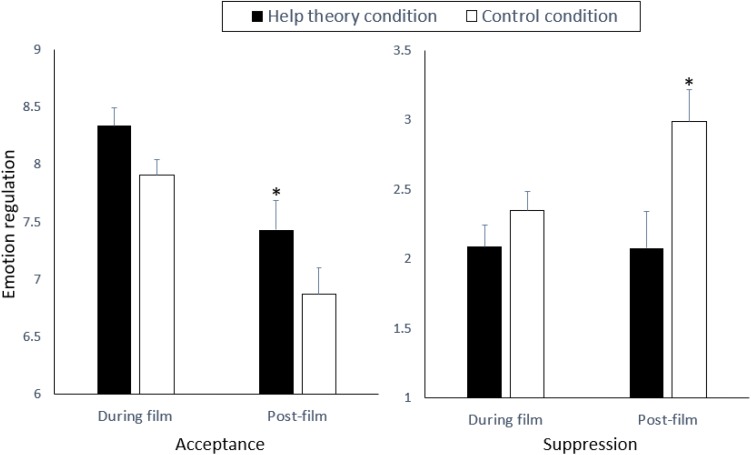
Mean ratings of emotional acceptance and experiential suppression during and after a distressing film by participants in the Help Theory and control conditions in Study 2. Error bars represent ±1 SE. Asterisks represent statistically significant comparisons (*p* < 0.05).

Figure 2 shows mean acceptance and experiential suppression by condition over time. For each emotion regulation strategy, we conducted a 2 (time: distressing film, post-film rest period) × 2 (condition) mixed model ANOVA. For acceptance, the results showed main effects of time and condition. Overall, participants reported accepting their feelings more during the distressing film than during the post-film rest period, *F*(1,151) = 32.59, *p* < 0.001, η^2^ = 0.18. Participants in the Help Theory condition accepted their emotions more than did those in the control condition, *F*(1,151) = 4.77, *p* < 0.05, η^2^ = 0.03. For experiential suppression, the results showed an interaction between time and condition, *F*(1,151) = 4.55, *p* < 0.05, η^2^ = 0.03. During the distressing film, participants did not differ by condition in their use of suppression, *t*(1,151) = 0.87, *p* = 0.39. After the film, however, participants in the Help Theory condition suppressed their emotional experience less than did those in the control condition, *t*(1,151) = 2.85, *p* = 0.005, *d* = 0.38. Perhaps because the inhumane acts depicted in the film *Cry Freedom* did not lend themselves to reappraisal, participants reported little reappraisal during or after the film (all means < 2.18), and reports of engaging in reappraisal did not differ between the Help Theory and control conditions (*t*s < 1.42, *p*s > 0.16).

### Discussion

In Study 2, we manipulated participants’ views about the value of emotion. Although self-reported negative emotion did not differ between conditions, participants in the Help Theory condition showed greater physiological reactivity during the distressing film than did those in the control condition. Yet, physiological reactivity decreased significantly from the end of the distressing film to the end of the post-film period for participants in the Help Theory condition, suggesting recovery. Physiological reactivity did not decrease after the distressing film for participants in the control condition. Participants in the Help Theory condition also reported greater acceptance of their emotional response than did control participants, engaged in less experiential suppression after the film, and reported that the film had less effect on their current mood after the film.

Experiments provide control and the opportunity to assess causal effects but can be subject to demand characteristics. However, the current findings do not correspond to the pattern of differences likely to result from experimenter demand. If participants were attempting to respond as they believed the experimenter preferred, the Help Theory and control conditions would likely differ in self-reported emotion but not in physiological reactivity. Instead, self-reported emotion did not differ between conditions, but participants in the Help Theory condition showed greater SCL during the distressing film and a decrease in SCL after the film. In addition, social desirability was not associated with SCL levels during or after the distressing film for participants in either condition (*p*s > 0.10). Finally, the greater acceptance reported by participants in the Help Theory condition is consistent with the results of Study 1 which did not manipulate beliefs about emotion. Thus, the results of Study 2 suggest that believing that emotion has value promotes acceptance of emotional experience and physiological and mood recovery after distressing events.

## General Discussion

Debates about whether emotion is adaptive or maladaptive predate Plato and are still salient in Western media and discourse today (Lutz, 1986; Karnaze and Levine, 2018). Recently, researchers have also begun to explore lay people’s views about the functionality of specific emotional states or features such as stress and physiological arousal. However, people’s theories about the overall functionality of emotion are not well understood. This investigation examined how lay beliefs about the functionality of emotion shape people’s emotional experience, the strategies they adopt to regulate emotion, and their recovery after distressing events. Study 1 described the development and testing of a measure of people’s theories about the extent to which emotion is a help or a hindrance. Participants who more strongly endorsed a Help Theory reported greater wellbeing, emotional acceptance, and use of reappraisal to regulate emotion. Participants who more strongly endorsed a Hinder Theory reported less wellbeing and more expressive suppression and use of substances to cope with stress. The results of Study 2 showed that encouraging participants to view emotion as helpful promoted emotional acceptance and recovery from a physiologically arousing negative experience.

### Assessment and Correlates of Beliefs About the Overall Functionality of Emotion

The results of Study 1 support the reliability and validity of the new 8-item instrument, the Help and Hinder Theories about Emotion Measure (HHTEM), which assesses an individual’s beliefs about the overall functionality of emotion. Help Theory scores showed acceptable alpha but Hinder Theory scores showed low alpha. However, scales that are shorter and have less redundant items tend to have lower alphas. Therefore, we also assessed the average item intercorrelations, which indicated that the items within each construct were moderately correlated. Help and Hinder Theory showed less stability over time, but fell within the range of test-retest correlations for convergent measures. Because beliefs about emotion should rely on memory for past emotional experiences, which can be shaped by current feelings or appraisals of past experiences (Levine et al., 2018), in future work it will be important to encourage participants to think about emotions more generally, rather than in relation to their current circumstances.

Help and Hinder Theories converged with several constructs about emotional experience in expected ways. Consistent with our previous findings (Karnaze and Levine, 2018), Help Theory endorsement was associated with experiencing emotion with greater, rather than less, intensity. The more participants endorsed a Help Theory, the more they viewed positive and negative emotion as being meaningful and helpful, and the more they reported attending to and following their feelings. People who more strongly endorsed a Hinder Theory tended to ignore their positive feelings, but reported both ignoring and attending to negative feelings, perhaps indicating ambivalence toward their unpleasant emotions. Hinder Theory endorsement was not related to emotional intensity. Hinder Theory endorsement was associated with viewing both positive and negative feelings as pointless and with viewing positive feelings as disruptive. Participants who more strongly endorsed a Hinder Theory tended to ignore positive feelings but reported both ignoring and attending more to negative feelings. Thus, people who view emotion as a hindrance may attempt to ignore negative feelings but find themselves nonetheless under their sway. Importantly, Help and Hinder Theories did not merely reflect people’s tendency to approach rewarding experiences or avoid punishment, to place less or greater value on cognition, or to present themselves in a positive manner.

Participants who viewed emotion as more helpful also reported using regulatory strategies that are often adaptive: reappraisal, acceptance, planning, and positive reframing. In contrast, Hinder Theory endorsement was related to engaging in expressive suppression and using substances to cope with stress. Together, these findings suggest that people who view emotion as helpful tend to engage in regulation strategies that involve reflecting on their emotional experience. In contrast, those who view emotion as harmful engage in strategies directed toward ridding themselves of their feelings or altering feelings without addressing their underlying causes. Consistent with findings that using adaptive emotion regulation strategies promotes wellbeing (e.g., Gross and John, 2003), participants who viewed emotion as more helpful also reported more happiness and social support and tended to be more satisfied with life. Participants who more strongly viewed emotion as a hindrance reported less happiness and were more likely to report clinically significant depressive symptoms. Thus, Study 1 revealed theoretically expected associations between people’s beliefs about the functionality of emotion and their emotional experience, regulatory strategies, and wellbeing.

Developing and testing the HHTEM is a critical step toward determining whether viewing emotion overall as adaptive gives people advantages by predisposing them to feel better about their emotional reactions, better regulate their emotions, receive more social support, and thus experience greater wellbeing over time. It also makes the unique contribution of tapping beliefs about the harmful nature of emotion and their correlates. However, correlational data cannot speak to the causal direction of these associations. Therefore, in Study 2, we experimentally manipulated the extent to which participants endorsed a Help Theory about emotion.

### Effects of Lay Theories About Whether Emotion Helps

The results of Study 2 showed that people could be encouraged to view both positive and negative emotion as helpful for reasoning and wellbeing. Moreover, manipulating participants’ beliefs about the functionality of emotion affected their emotional acceptance during a distressing film and recovery afterward. Overall, participants perceived the events of the distressing film as very upsetting, and self-reports of negative emotion did not differ as a function of condition. Yet, relative to controls, participants in the Help Theory condition reported greater emotional acceptance. They also showed higher skin conductance, a marker of sympathetic nervous system activation, during the distressing film than did participants in the control condition. SCL and subjective emotional experience are not always correlated (Mauss et al., 2005) and encouraging participants in the Help Theory condition to value emotion may have led them to empathize more with the protagonists and feel threatened, resulting in sympathetic nervous system arousal. After the distressing film, however, participants in the Help Theory condition reported suppressing their negative feelings less, and reported their current mood was affected less by the film, than control participants. In addition, those in the Help Theory condition, but not in the control condition, showed a decrease in skin conductance in the period after the film, suggesting recovery. Thus, believing that emotion has value promoted emotional acceptance and physiological and mood recovery after a distressing experience.^6^

These findings suggest that, when people encounter distressing situations, those who value emotion allow themselves to more fully experience their emotional reactions in the moment. Because they value emotion, they may feel less distressed by their reaction, allowing them to recover quickly. Future research could test this by examining personal events that are physiologically arousing (e.g., a stress test) and measuring physiological recovery over a longer time (e.g., cortisol reactivity). Even if people have intense subjective emotional and physiological responses to distressing events, their ability to recover from such events can have subsequent mental and physical health benefits (e.g., Leger et al., 2018).

### Limitations and Future Research Directions

In Study 1, Cronbach’s alpha for the Help scale was acceptable (0.74), but Cronbach’s alpha for the Hinder Scale (0.64) only minimally met the threshold considered acceptable for an *ad hoc* scale. Thus, modification to improve the internal consistency of items is needed before the Hinder Scale can be recommended for use in future research. In addition, the test-retest correlation for the combined Help and Hinder Theory scale was low (*r* = 0.46). This raises the question of how stable beliefs about the functionality of emotion are. Future research is needed to ensure that researchers can measure global beliefs about the functionality of emotion irrespective of current events that may evoke transient positive or negative emotional reactions. Beliefs about the functionality of emotion may also change across developmental periods. We examined lay theories among samples of college students. It will be important to examine endorsement of these theories among older adults who tend to value positive emotional experiences and reappraise or avoid negative emotional experiences (Carstensen et al., 2003). In Study 2, encouraging a Help Theory did not increase reappraisal of the violent and unjust historical events depicted in the film. Future research should also examine whether promoting a Help Theory about emotion leads people to engage in reappraisal in circumstances that lend themselves to the use of this strategy. Future research should also assess the long-term implications of lay theories about emotion for wellbeing. For example, researchers could encourage a Help Theory before a major life transition, such as a school or career change, and assess downstream links to adjustment, social support, and wellbeing. Importantly, feeling satisfied with relationships and life in general could promote a Help Theory about emotion, so it is important to look at whether a Help Theory predicts long-term support and wellbeing during periods of transition. Further research on viewing emotion overall as a hindrance is also important. If people who regard their emotions as generally harmful can learn to recognize the important functions emotions fulfill, they may feel better over time because they are less alarmed by their responses to life events. Interventions designed to encourage viewing emotion as adaptive, combined with training in emotion regulation, could help people be more strategic and effective in selecting emotion regulation strategies in daily life, rather than trying to mask, numb, ignore, or eradicate undesired feelings.

Finally, given the importance of lay theories of the functions of emotion, it will be important to explore how these theories develop, and how they relate to the development of personality traits and decision-making strategies (e.g., people’s tendency to “trust their gut,” openness to experience, neuroticism^7^) (for related approaches, see Walle and Campos, 2012; Dweck, 2017) as well as culture. The samples were relatively diverse in terms of race-ethnicity, and we did not find gender or ethnicity differences in these studies. However, in past research, we found that men tended to view emotion as more hindering than women, and that Asian and Hispanic participants viewed emotion as more hindering than White participants (Karnaze and Levine, 2018). The role of culture in shaping lay theories about the functionality of emotion is an important issue for future research, as cultures that tend to value individual expression may view emotion as more helpful than cultures that prioritize the needs of the social group.

## Conclusion

In conclusion, the results of the current investigation show that people’s beliefs about the value of emotion matter. Taken together, the new HHTEM and these studies demonstrate that it is advantageous for people to view emotion overall as functional. Even if a specific emotional experience is not helpful in a situation, viewing emotion overall as adaptive predisposes people to be more accepting and less distressed by their own emotional reactions, better regulate their emotions, receive more social support, and experience greater wellbeing over time. The HHTEM also makes the unique contribution of tapping beliefs that emotion is harmful overall, providing evidence about the ways that holding a negative view of emotion can put people at risk.

## Data Availability Statement

The datasets generated for this study are available on request to the corresponding author.

## Ethics Statement

All studies were approved by, and carried out in accordance with the recommendations of, the Institutional Review Board of the University of California, Irvine. All subjects in Study 2 provided written informed consent. In accordance with the national legislation and institutional requirements, written consent was not required for the participation in Study 1.

## Author Contributions

MK and LL conducted the research and wrote the manuscript.

## Conflict of Interest

The authors declare that the research was conducted in the absence of any commercial or financial relationships that could be construed as a potential conflict of interest.

## Footnotes

1 Individuals who do not view emotion as helpful, and particularly those who view emotion as a hindrance, may use a range of strategies to inhibit and avoid emotion. Some strategies, such as suppressing emotion-expressive behavior and engaging in repressive coping, have been shown to increase sympathetic activation of the cardiovascular system (e.g., Roberts et al., 2008). Other strategies, such as avoiding emotional situations, attentional disengagement, self-distancing, and dissociation have been shown to decrease sympathetic activation (e.g., Fraley and Shaver, 1997; Sheppes et al., 2009; Hetzel-Riggin and Wilber, 2010; Dewe et al., 2016; Kross and Ayduk, 2017). Thus, even though individuals who endorse a Hinder Theory are more likely to engage in expressive suppression, we expected those who view emotions as helpful to show greater physiological arousal because they attend to and permit the progression of their emotional reactions.

2 The 15 preliminary Help and Hinder Theory items in Study 1, and the factor analysis of those items, are available online at https://osf.io/4vkfq/in Supplementary Tables 2, 3.

3 In addition to the measures listed, the three questionnaires in Study 1 included exploratory questions (e.g., concerning health, academics) that were not the focus of the current investigation and additional measures that are listed online in Supplementary Text.

4 In Study 2, we pilot-tested a Hinder Theory induction (*N* = 31). The instructions were identical to those for the Help Theory induction except that they emphasized how emotions were harmful rather than helpful in daily life and in the transition to life as a college student. Preliminary analyses showed that instructions designed to encourage viewing emotion as a hindrance did not increase Hinder Theory endorsement, or decrease Help Theory endorsement, relative to the control condition (*p*s > 0.41). Therefore, this investigation focused on increasing Help Theory endorsement.

5 Preliminary analyses showed that ratings of subjective emotion and emotion regulation in Study 2 did not differ significantly when neutral film SCL or violence rating were included as covariates, so these analyses are presented without covariates. Mean values for subjective emotion, by time and condition, are available online in Supplementary Table 10. Mean values for emotion regulation, by time and condition, are available online in Supplementary Table 11. Mean values for skin conductance, by time and condition, are displayed online in Supplementary Figure 1.

6 The help theory condition differed from the control condition in four ways: Participants in the Help Theory condition showed greater skin conductance during the distressing film, more acceptance during the distressing film, less experiential suppression during the post-film rest period, less effect of the distressing film on mood. One potential objection to our interpretation of these findings is that these differences between the help and control conditions could have been due to the use of an induction that discussed emotion rather than to encouraging participants to view emotion as helpful *per se*. However, as explained in Footnote 3, we initially piloted participants with a Hinder Theory induction. Preliminary analyses showed no differences between the Hinder Theory condition and the control condition for any of the four variables (*p*s ranged from 0.07 to 0.92). These results suggest that the differences found between the Help Theory and control conditions were due to inducing participants to view emotion as helpful rather than to use of an experimental procedure that directed participants’ attention to emotion.

7 As we noted in the online Supplementary Materials (https://osf.io/4vkfq/), participants also completed The Big Five Personality Inventory (John and Srivastava, 1999), though this measure was not a focus of the present investigation. Supplementary Table 9 shows how Help and Hinder Theory endorsement was related to personality traits.

## Appendix

**TABLE 7:** Items and instructions for Help and Hinder Theories about Emotion (HHTEM) Scale.

Instructions	People can experience many different kinds of emotion, such as anger, disgust, sadness, fear, joy, love, pride, and awe. We want to know what you think about emotion overall. Considering emotion overall, how often is each statement below true? 0 = Almost Never 1 2 = Sometimes 3 4 = Almost Always
Help Theory Items	
	(1) Emotion helps people focus on what’s important
	(2) Emotion is a source of wisdom
	(3) Emotion helps people know what’s beneficial or harmful
	(4) Emotion is a strength that humans have
Hinder Theory Items	
	(1) Emotion distracts people from what’s important
	(2) Emotion makes life confusing
	(3) Emotion clouds judgment about right and wrong
	(4) Emotion is a weakness humans have
