# Supplementary material for: Lay Theories About Whether Emotion Helps or Hinders: Assessment and Effects on Emotional Acceptance and Recovery From Distress
Source: Front Psychol. 2020 Feb 18;11:183. doi: 10.3389/fpsyg.2020.00183 (PMC7040216; doi:10.3389/fpsyg.2020.00183)
Supplement: Supplementary file 1 [file Data_Sheet_1.pdf]

Supplemental Materials

Supplemental Table 1

Study 1: Initial 36 Items Used to Assess Help and Hinder Theories about Emotion, Numbered in Order of Presentation

Dimension	Help Theory Item	Hinder Theory Item
Emotion is motivating/disruptive	7- Emotion helps people achieve their goals<br>5-Emotion helps people solve problems<br>32-Emotion gives people energy to succeed<br>29-Emotion helps people focus on what's important<br>6-Emotion helps people avoid harm<br>31-Emotion motivates people's plans to succeed	20-Emotion prevents people from achieving their goals<br>19-Emotion creates problems for people<br>8- Emotion drains people's energy<br>17- Emotion distracts people from what's important<br>30-Emotion gets people into trouble<br>18-Emotion disrupts people's plans to succeed
Emotion is informative/irrational	11- Emotion is a source of wisdom<br>9- A person's emotions reveal what they value<br>27- Emotion helps people make good decisions<br>10-People's emotions help them know what's right and wrong<br>28-People need emotion to know what's beneficial or harmful<br>12-Emotion helps people see situations clearly	26-Emotion is a source of foolishness<br>25-When people feel emotion they cannot be objective<br>15-Emotion leads people to make poor decisions<br>16- Emotion clouds judgment about right and wrong<br>14-Emotion makes it hard to know what's beneficial or harmful<br>13-Emotion interferes with rational thought
Emotion is essential for life satisfaction/ a threat to life satisfaction	4- Emotions have value<br>34-Emotion is a strength that humans have<br>21- People need emotion to live a fulfilling life<br>2- Emotion helps people connect to others<br>24-Emotion helps people have good relationships<br>22-Emotion gives life meaning	36-Emotions serve no purpose<br>23-Emotion is a weakness that humans have<br>3- Emotion prevents people from living a fulfilling life<br>33-Emotion alienates people from others<br>35-Emotion interferes with having good relationships<br>1-Emotion makes life confusing

Supplemental Table 2

Study 1 Pilot Data: Mean Endorsement of 15 Preliminary Help and Hinder Theory Items, and Percentage of Participants Who Reported Rating Each Item Based on Emotion Overall, Mostly Positive Emotion, or Mostly Negative Emotion

	Endorsement		Percentage of Participants		
Lay Theory Item	*M*	*SD*	Emotion Overall	Mostly Positive	Mostly Negative
Help Theory					
1. People need emotion to know what's beneficial and harmful	3.34	0.79	77.3%	10.5%	6.5%
2. People's emotions help them know what's right and wrong	3.54	0.74	74.5%	12.1%	7.7%
3. A person's emotions reveal what they value	3.89	0.73	68.0%	23.1%	3.2%
4. Emotion helps people see situations clearly	2.84	0.85	58.7%	24.7%	11.8%
5. Emotion is a source of wisdom	2.84	0.85	54.7%	36.0%	3.6%
6. Emotion helps people focus on what's important	3.28	0.68	55.1%	35.6%	3.6%
7. Emotion is a strength that people have	3.77	0.84	49.4%	41.7%	3.2%
8. People need emotion to live a fulfilling life	4.08	0.83	49.0%	43.3%	2.0%
9. Emotion helps people avoid harm	2.39	0.69	40.5%	32.0%	21.9%
Hinder Theory					
1. Emotion makes it hard to know what's beneficial or harmful	3.06	0.73	67.6%	7.3%	19.8%
2. Emotion clouds judgment about right and wrong	3.14	0.61	55.9%	8.9%	29.6%
3. Emotion distracts people from what's important	2.98	0.60	49.4%	10.1%	34.8%
4. Emotion is a weakness that people have	2.57	1.02	48.2%	6.5%	39.7%
5. Emotion makes life confusing	2.36	0.61	40.1%	17.0%	47.4%
6. Emotion is a source of foolishness	2.39	0.86	45.7%	10.1%	38.5%

Note. Means and standard deviations are based on ratings of the 15 items on a scale from 0 (*almost never*) through 2 (*sometimes*) to 4 (*almost always*). After rating all items, participants indicated whether, when rating each item, they had been thinking about emotion overall, mostly about positive emotion, or mostly about negative emotion.

Supplemental Table 3

Study 1 Pilot Data: Results of an Exploratory Factor Analysis of 15 Initial Help and Hinder Items showing Item Loadings on Emergent Factor 1 and Factor 2

	Factor Loading	
Lay Theory and Item	*1*	*2*
Help Theory		
People need emotion to know what's beneficial or harmful	.662	.203
People's emotions help them know what's right and wrong	.621	.248
A person's emotions reveal what they value	.391	.325
Emotion helps people see situations clearly	.576	.169
Emotion is a source of wisdom	.682	.266
Emotion helps people focus on what's important	.650	.297
Emotion is a strength that humans have	.597	.151
People need emotion to live a fulfilling life	.502	.294
Emotion helps people avoid harm	.404	.066
Hinder Theory		
Emotion makes it hard to know what's beneficial or harmful	-.396	.613
Emotion clouds judgment about right and wrong	-.313	.704
Emotion distracts people from what's important	-.429	.490
Emotion is a weakness that humans have	-.480	.361
Emotion makes life confusing	-.368	.497
Emotion is a source of foolishness	-.615	.198

Supplemental Table 4

Study 1: Internal Reliability and Test-Retest Reliability between Time 1 and Time 3

Variable	α	~4 Week Test-Retest Correlation
1. Help Theory	.74	.48***
2. Hinder Theory	.64	.51***
3. PA Valuation	.75	.85***
4. NA Valuation	.64	.28***
5. PA Utility	.85	.65***
6. NA Utility	.84	.57***
7. Following PA	.75	.60***
8. Ignoring PA	.75	.57***
9. Following NA	.70	.67***
10. Ignoring NA	.74	.56***

Note. Measures assessed were Help and Hinder Theories, Valuation of Positive and Negative Affect Scales, Perceived Utility of Positive and Negative Affect Scales, and Following Affect Scales Test.

***$p < .001$.

Supplemental Table 5

Study 1 Correlations of HHTEM Subscales with Convergent Measures

Variable	1	2	3	4	5	6	7	8	9	10
1. Emotion helps	–	-.01	.20**	.05	.25***	.15*	.29***	.17**	-.23**	-.17**
2. Emotion hinders		–	-.11	.01	-.01	.14*	-.01	.20**	-.23**	.36***
3. Positive Affect Valuation			–	-.27***	.51***	.03	.56***	.01	-.52***	.03
4. Negative Affect Valuation				–	-.22***	-.10	-.17**	-.10	.17**	-.15**
5. Positive Affect Utility					–	.21***	.58***	-.02	-.33***	.09
6. Negative Affect Utility						–	.07	.25***	-.01	-.05
7. Attention to Positive Feelings							–	-.11	-.50***	.07
8. Attention to Negative Feelings								–	.05	-.02
9. Ignoring Positive Feelings									–	.29***
10. Ignoring Negative Feelings										–

Note. $*p < .05$. $**p < .01$. $***p < .001$

Supplemental Table 6

Study 1 Correlations of HHTEM Subscales with Divergent Measures

Variable	Correlations					
	1	2	3	4	5	6
1. Emotion helps	–	-.02	.06	.01	.04	-.12
2. Emotion hinders		–	-.07	.06	.01	-.05
3. Need for cognition			–	-.26***	.19**	.15*
4. Approach motivation				–	.56*	.05
5. Avoidance motivation					–	.24***
6. Social desirability						–

Supplemental Table 7

Study 1 Bootstrap Correlations of HHTEM Subscales with Emotion Regulation and Coping Strategies at Time 1

Variable	1	2	3	4	5	6	7	8	9	10	11
1. Emotion helps	–	-.01	.18**	-.01	.08	.18	.18**	.16**	.21***	.22***	.12
2. Emotion hinders		–	.01	.17**	-.01	.01	.03	.03	.02	.10	.25***
3. Cognitive reappraisal			–	-.02	.35***	.41***	.37***	.52***	.17**	.12	-.09
4. Expressive suppression				–	-.08	-.15*	-.08	-.10	-.30***	-.31***	.15*
5. Acceptance					–	.33***	.38***	.34***	.23***	.18**	-.08
6. Active coping						–	.63***	.36***	.11	.15*	-.03
7. Planning							–	.28***	.15*	.11	-.08
8. Positive reframing								–	.26***	.25***	.02
9. Instrumental social support									–	.77***	-.05
10. Emotional social support										–	.07
11. Substance use											–

Note. *$p < .05$. **$p < .01$. ***$p < .001$.

Supplemental Table 8

Study 1 Bootstrap Correlations of HHTEM Subscales with Emotion Regulation and Coping Strategies at Time 2

Variable	1	2	3	4	5	6	7	8	9	10	11
1. Emotion helps	–	-.07	.18**	-.01	.17*	.13†	.16*	.15	.21**	.28***	-.02
2. Emotion hinders		–	.01	.08	.07	-.02	.07	.03	.03	.02	.18**
3. Cognitive reappraisal			–	.04	.37***	.39***	.34***	.55***	.20**	.22**	-.12
4. Expressive suppression				–	-.05	-.10	-.01	-.13	-.38***	-.40***	.05
5. Acceptance					–	.42***	.47***	.37***	.18**	.21**	-.02
6. Active coping						–	.60***	.40***	.23**	.25***	-.05
7. Planning							–	.40***	.26***	.24***	-.01
8. Positive reframing								–	.27***	.31***	.03
9. Instrumental social support									–	.86***	.01
10. Emotional social support										–	-.38***
12. Substance use											–

†*p* = .05. *p < .05. **p < .01. ***p < .001.*

Supplemental Table 9

Study 1 Correlations of HHTEM Subscales with Personality Dimensions

Variable	1	2	3	4	5	6	7
1. Emotion helps	–	-.02	.10	.21***	.20**	.06	.20**
2. Emotion hinders		–	-.01	-.20**	-.06	.15*	-.06
3. Extraversion			–	.17**	.28***	-.42***	.17**
4. Agreeableness				–	.41***	-.25***	.27***
5. Conscientiousness					–	-.30***	.22***
6. Neuroticism						–	-.09
7. Openness							–

Note. *$p < .05$. **$p < .01$. ***$p < .001$.

Supplemental Table 10

Study 2: Mean Ratings of Positive and Negative Emotion by Condition during the Neutral Film, during the Distressing Film, and After the Distressing Film

	Help Theory Condition	*Control Condition*
Emotion Variable	*M* (*SD*)	*M* (*SD*)
Neutral film		
Positive emotion	4.91_a (1.72)	2.07_a (2.07)
Negative emotion	1.70_a (0.85)	1.80_a (0.91)
Distressing film		
Positive emotion	3.66_a (1.22)	3.55_a (1.53)
Negative emotion	6.02_a (1.19)	6.17_a (1.38)
Mood affected by distressing film	7.15_a (1.82)	7.12_a (2.01)
Four-minute rest period		
Positive emotion	2.28_a (1.50)	2.58_a (1.48)
Negative emotion	2.68_a (1.78)	2.93_a (1.73)
Mood affected by distressing film	5.08_a (2.57)	5.85_b (2.11)

Note. Means with different subscripts differed at $p < .05$.

Supplemental Table 11

Study 2: Mean Ratings of Emotion Regulation, During and After a Distressing Film, by Condition

	Help Theory Condition	*Control Condition*
Emotion Regulation	*M* (*SD*)	*M* (*SD*)
Distressing Film		
Acceptance	8.34_a (0.99)	7.91_b (1.45)
Experiential Suppression	2.09_a (1.78)	2.35_a (1.87)
Cognitive Reappraisal	1.93_a (1.50)	2.09_a (1.73)
Four-Minute Rest Period		
Acceptance	7.43_a (2.20)	6.87_a (2.05)
Experiential Suppression	2.08_a (1.60)	2.99_b (2.21)
Cognitive Reappraisal	1.78_a (1.54)	2.17_a (1.82)

Note. Means with different subscripts differed at $p < .05$.

Panel A

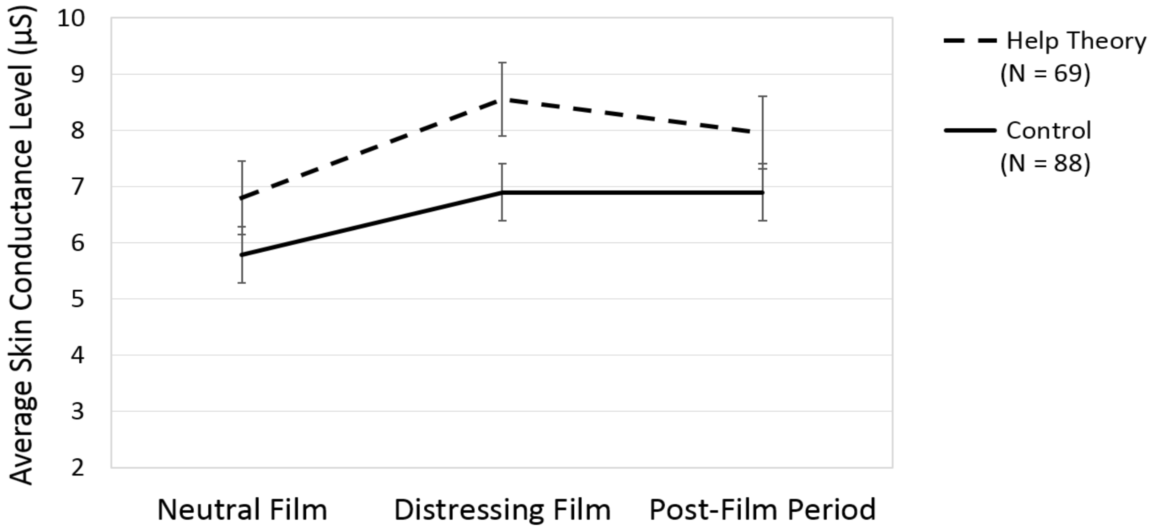

Panel B

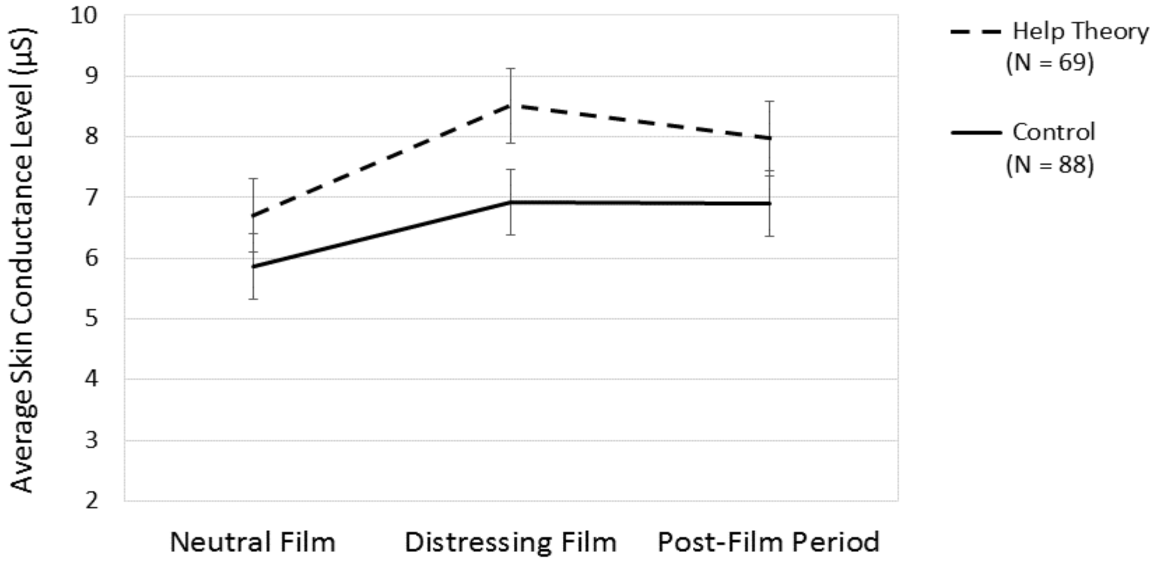

Supplemental Figure 1. Skin conductance over time by condition in Study 2. Panel A shows unadjusted mean SCL over time. Panel B shows mean SCL over time, adjusted for the violence rating for the distressing film. Bars represent +/-1 SE.

Supplemental Text:

Additional Measures on the Study 1 Questionnaires

In addition to the measures listed in the main text for Study 1, participants answered questions that were not expected to relate specifically to beliefs about the functionality of emotion, and were not the focus of this investigation. The additional measures included in each questionnaire are listed below.

Time 1 Questionnaire

In addition to the measures listed in the text, on the first questionnaire, participants answered questions concerning the previous night's sleep quality, exercise habits, the day's current stress level, and their experiences in college. They also completed exploratory questions about the degree to which they tried to regulate how they felt about their schoolwork, responsibilities outside of school, relationships, and their life overall. Participants reported how many sick days they took in the last three months, and completed the perceived health subscale of the Medical Outcomes Study general short form Health Survey (Stewart, Hays, & Ware, 1988). Participants answered acculturation questions about where they were born, the languages they spoke, and their parents' demographics information. Participants also answered exploratory questions concerning emotion regulation and beliefs about emotions that do not represent help or hinder theories.

Participants also completed the following five measures. (1) They completed a modified version of the Implicit Beliefs about Emotion scale (De Castella et al., 2013; based on Tamir et al., 2007) to assess the degree to which they believed their emotions were fixed (reverse-scored), or that they could change or control their emotions (α = 0.77), using a scale from 1 (*strongly disagree*) to 5 (*strongly agree*). (2) Participants completed the trait anxiety scale of the State-Trait anxiety inventory (Spielberger, 2010) by rating how they general felt (e.g., "I feel nervous") using a scale from 1 (*Almost never*) to 4 (*Almost always*). (3) Participants completed

The Big Five Personality Inventory (John & Srivastava, 1999) assessed the personality dimensions of agreeableness, conscientiousness (α = 0.61), extraversion (α = 0.85), neuroticism (α = 0.81), and openness to experience (α = 0.72). Participants rated the extent to which several statements described themselves, using a scale from 1 (*Disagree strongly*) to 5 (*Agree strongly*). (4) Participants rated the Single-Item Self Esteem Scale (Robins, Hendin, & Trzesniewski, 2001) using a five-point scale of agreement. (5) Participants also completed The Toronto Empathy Questionnaire (Spreng, McKinnon, Mar, & Levine, 2009), which assessed the ability to perceive others' feeling states, with 16 statements (e.g., "I can tell when others are sad even when they do not say anything") using a scale from 0 (*never*) to 4 (*always*; α = .62).

Time 2 Questionnaire

In addition to the measure listed in the text, on the second questionnaire, participants reported their baseline mood, sleep quality, exercise habits, stress, and grade point average. They also completed a measure of how often they ruminate (Treynor, Gonzalez, & Nolen-Hoeksema, 2003).

Time 3 Questionnaire

In addition to the measures listed in the text, on the third questionnaire, participants completed a measure of familism values (Sabogal Marín, Otero-Sabogal, Marín, & Perez-Stable, 1987), a measure of trait acceptance (Baer, Smith, & Allen, 2004), and an inventory of stressful life events. Participants also completed questions about their religiosity and political ideology.

References Cited in Supplemental Materials

Baer, R. A., Smith, G. T., & Allen, K. B. (2004). Assessment of mindfulness by self-report: The Kentucky Inventory of Mindfulness Skills. *Assessment*, *11*, 191-206.

DeCastella, K., Goldin, P., Jazaieri, H., Ziv, M., Dweck, C.S., & Gross, J.J. (2013). Beliefs about emotion: Links to emotion regulation, well-being, and psychological distress. *Basic and Applied Social Psychology, 35,* 497-505.

John, O. P., & Srivastava, S. (1999). The Big Five trait taxonomy: History, measurement, and theoretical perspectives. *Handbook of Personality: Theory and research*, *2*, 102-138.

Mitmansgruber, H., Beck, T. N., Höfer, S., & Schüßler, G. (2009). When you don't like what you feel: Experiential avoidance, mindfulness and meta-emotion in emotion regulation. *Personality and Individual Differences*, *46*, 448–453.

Robins, R. W., Hendin, H. M., & Trzesniewski, K. H. (2001). Measuring global self-esteem: Construct validation of a single-item measure and the Rosenberg Self-Esteem Scale. *Personality and Social Psychology Bulletin*, *27,* 151-161.

Sabogal, F., Marín, G., Otero-Sabogal, R., Marín, B. V., & Perez-Stable, E. J. (1987). Hispanic familism and acculturation: What changes and what doesn't?. *Hispanic Journal of Behavioral Sciences*, *9*, 397-412.

Spielberger, C.D. (2010) State-Trait Anxiety Inventory. *Corsini Encyclopedia of Psychology*. John Wiley & Sons, Inc., Hoboken.

Spreng, McKinnon, Mar, & Levine, B. (2009). The Toronto Empathy Questionnaire: Scale development and initial validation of a factor-analytic solution to multiple empathy measures. *Journal of Personality Assessment*, *91*, 62-71.

Stewart, A. L., Hays, R. D., & Ware, J. E. (1988). The MOS short-form general health survey: reliability and validity in a patient population. *Medical Care*, *26*, 724-735.

Tamir, M., John, O. P., Srivastava, S., & Gross, J. J. (2007). Implicit theories of emotion: Affective and social outcomes across a major life transition. *Journal of Personality and Social Psychology*, *92*, 731-744.

Treynor, W., Gonzalez, R., & Nolen-Hoeksema, S. (2003). Rumination reconsidered: A psychometric analysis. *Cognitive Therapy and Research*, *27,* 247-259.
